# Improving the design of studies evaluating the impact of diagnostic tests for tuberculosis on health outcomes: a qualitative study of perspectives of diverse stakeholders

**DOI:** 10.12688/wellcomeopenres.15551.1

**Published:** 2019-11-21

**Authors:** Eleanor A. Ochodo, Selvan Naidoo, Samuel Schumacher, Karen Steingart, Jon Deeks, Frank Cobelens, Patrick M. Bossuyt, Taryn Young, Mark P. Nicol

**Affiliations:** 1 Centre for Evidence-based Health Care, Department of Global Health, Faculty of Medicine and Health Sciences, Stellenbosch University, Cape Town, Western Cape, 8000, South Africa; 2 Tuberculosis department, Campus Biotech, Foundation for Innovative New Diagnostics, Geneva, 1202, Switzerland; 3 Clinical sciences, Liverpool School of Tropical Medicine, Liverpool, L3 5QA UK, UK; 4 NIHR Birmingham Biomedical Research Centre, University Hospitals Birmingham NHS Trust and University of Birmingham; and Test Evaluation Research Group, Institute of Applied Health Research, University of Birmingham, Edgbaston, Birmingham, B15 2TT, UK; 5 Department of Global Health and Amsterdam Institute for Global Health and Development, Amsterdam University Medical Centers, Amsterdam, 1105 BP, The Netherlands; 6 Department of Clinical Epidemiology, Biostatistics and Bioinformatics, Amsterdam Public Health research institute, Amsterdam University Medical Centers, Amsterdam, 1105 AZ, The Netherlands; 7 School of Biomedical Sciences, Faculty of Health and Medical Sciences, University of Western Australia, Perth, WA 6009, Australia

**Keywords:** Tuberculosis, Qualitative research, Perspectives, Impact, TB tests impact, TB diagnostic tests

## Abstract

**Background: **Studies evaluating the impact of Xpert MTB/RIF testing for tuberculosis (TB) have demonstrated varied effects on health outcomes with many studies showing inconclusive results. We explored perceptions among diverse stakeholders about studies evaluating the impact of TB diagnostic tests, and identified suggestions for improving these studies.

**Methods:** We used purposive sampling with consideration for differing expertise and geographical balance and conducted in depth semi-structured interviews. We interviewed English-speaking participants, including TB patients, and others involved in research, care or decision-making about TB diagnostics. We used the thematic approach to code and analyse the interview transcripts.

**Results: **We interviewed 31 participants. Our study showed that stakeholders had different expectations with regard to test impact and how it is measured. TB test impact studies were perceived to be important for supporting implementation of tests but there were concerns about the unrealistic expectations placed on tests to improve outcomes in health systems with many influencing factors. To improve TB test impact studies, respondents suggested conducting health system assessments prior to the study; developing clear guidance on the study methodology and interpretation; improving study design by describing questions and interventions that consider the influences of the health-care ecosystem on the diagnostic test; selecting the target population at the health-care level most likely to benefit from the test; setting realistic targets for effect sizes in the sample size calculations; and interpreting study results carefully and avoiding categorisation and interpretation of results based on statistical significance alone. Researchers should involve multiple stakeholders in the design of studies. Advocating for more funding to support robust studies is essential.

**Conclusion: **TB test impact studies were perceived to be important to support implementation of tests but there were concerns about their complexity. Process evaluations of their health system context and guidance for their design and interpretation are recommended.

## Introduction

Tuberculosis (TB) continues to be a major public health burden. In 2018 it was estimated that about 10 million people developed TB disease, there were about half a million new cases of rifampicin-resistant TB, and 1.5 million deaths due to TB ^1^. The End TB strategy strives to reduce TB incidence by 80%, and TB mortality by 90% compared to 2015 levels. To facilitate progress towards these targets, the World Health Organization (WHO) recommends that countries aim to have 90% or more of TB patients diagnosed with WHO recommended rapid tests, and 90% or more of eligible patients treated with new recommended drugs by the year 2025 ^2^.

In order to improve TB case detection and rapid initiation of treatment, new rapid molecular diagnostic tests with reported high sensitivity and specificity and/or short-turnaround times, such as Xpert MTB/RIF and Xpert Ultra (the newest version) continue to be introduced to the market ^1, 3^. It is expected that accurate diagnosis and rapid initiation of treatment would improve downstream health outcomes such as morbidity and mortality.

However there is uncertainty about the effects of Xpert MTB/RIF on people-important outcomes, which include outcomes that directly reflect how an individual feels, functions or survives (patient health outcomes) ^4^, and outcomes that lie on the causal pathway through which a test can affect a patient's health, and thus predict patient health outcomes (surrogate or intermediate outcomes) ^5, 6^. Two recently published systematic reviews and meta-analyses of randomized trials suggest Xpert MTB/RIF likely reduces mortality ^7^ [odds ratio 0·88, 95% CI 0·68–1·14] and unfavorable treatment outcomes ^8^ [risk ratio 0.92, 95% CI 0.82–1.02] when compared to smear microscopy in adults with presumptive TB, but uncertainty in effect estimates was high. Pooled results in the meta-analyses suggested Xpert MTB/RIF did not affect time to diagnosis [hazard ratio 1·05, 95% CI 0·93–1·19] and time to treatment [hazard ratio 1·0, 0·75–1·32]. Confidence intervals were wide demonstrating large variation in estimates.

Randomized trials of diagnostic tests are typically considered the best way ^9^ to evaluate the effects or impact of interventions but these studies are challenging and their interpretation may not be straightforward ^10– 12^. A diagnostic test is evaluated as an element in a complex intervention, comprised of a sequence of interrelated events and decisions, all which vary across different study contexts ^12^. End users and other stakeholders may have different perspectives on the impact of diagnostic tests, outcome measures that matter, and how they should be evaluated. To our knowledge no systematic attempts to gather and analyze these perspectives have been published.

Qualitative research can help in understanding the complex phenomena at play and the varied perceptions of participants who are part of TB diagnostic test studies, and help to shed light on why and how these tests work in different contexts, and on how best to implement them ^13^ .

We explored perceptions of diverse stakeholders about studies evaluating the impact of TB diagnostic tests, and identified suggestions for improving these studies.

## Methods

### Study design

We conducted a qualitative study, using a phenomenological approach, that aimed to develop a complete description and understanding of human experiences and meanings, allowing findings to emerge from the data ^14^.

### Sampling and recruitment

Participants were purposively sampled from institutions known to our network and from other diagnostic forums such as the Stop TB New Diagnostics Working Group, and the Global Health Diagnostics community online (GHDonline). To source participants from GHDonline, we sent a general email to members on the platform inviting them to participate in the study. Invitation letters can be found in *Extended data:* Annex 1.

We only included English-speaking participants who had been involved in research, care, or decision making about both drug susceptible and drug resistant TB diagnosis. Considering that diagnostic tests need to function in a complex ecosystem of various users at various levels of the health care systems ^13^, we sampled diverse stakeholders. We considered maximum variation with regard to expertise (researchers, clinicians, laboratory workers, TB programme managers, guideline developers, policy makers, TB technical assistance and support agencies, funding agencies, patients, TB survivors and activists) and geographical location (from various low and high TB burden countries). We believed that a diverse group of stakeholders would give us a broader insight in designing, executing, interpreting and using TB studies for decision-making.

We sent out invitations to 60 potential participants, and interviewed only those who responded to, and accepted our invitation. We aimed to have a purposive sample of 30 participants in the study, since we anticipated that data saturation would have been reached with this number.

### Data collection

Data were collected through in-depth semi-structured interviews. We prepared an interview guide and tailored it to the different stakeholders we were interviewing ( *Extended data:* Annex 2). The topic guide was piloted by conducting mock interviews on three colleagues (not part of this project) from the Centre of Evidence-based health care in Stellenbosch University and modified based on the results of a pilot exercise.

Interviews were conducted by two researchers (EO [female] and SN [male] ^15^). EO has a medical background with further training in international health and clinical epidemiology. SN is an epidemiologist. Both EO and SN underwent an additional three-month training course in qualitative research methods and interview techniques.

Interviews were conducted in English via a conference call platform or by telephone. Teleconference interviews were conducted by EO with SN listening in and taking notes. Face to face interviews were conducted with patients in Khayelitsha community health clinic (Cape Town) by SN with the help of a professional interpreter who translated questions from English to the local language isiXhosa. Participant responses were then translated back to English.

There were no pre-established relationships between the interviewers and participants prior to the interviews. Participants were provided with information sheets and written consent forms prior to the interview; via Google Forms for teleconference interviews, and hard copies for face-to-face interviews. The content of consent forms was similar for non-patient participants and patients; however consent forms for patients were translated into the local language isiXhosa ( *Extended data:* Annex 3).

Interviews lasted between 30 to 45 minutes. Interview data were captured using a digital voice recorder. Interviews were transcribed for analysis, by a professional transcriber. All transcripts were audited for accuracy by the interviewer who conducted the interview. Names of participants did not appear on the transcripts. Transcripts were not returned to participants for corrections or clarification.

Data are stored electronically in password protected computers, and on secure online data storage platforms.

### Data analysis

Analysis of the interviews was done after data collection using thematic analysis ^16^. Two researchers EO and SN coded the interview transcripts together, discussing the codes and themes. EO and SN first familiarized themselves with the subject matter by listening to the audio tapes and reading the transcripts. The first transcript was coded independently and themes in the data were discussed. For feasibility reasons we decided to code subsequent transcripts together. Guided by the research questions, our analysis utilized deductive and inductive approaches grounded in the data. We did not apply line by line coding to every single line, but coded information that was relevant to our research question. We developed a broad set of codes, and modified or added to the codes as we read the transcripts. We coded the hard transcripts using the qualitative software Atlas ti. version 7.

We generated and merged similar codes to minimize duplication and improve readability and grouped the codes into sub-themes and themes in discussion with a senior author (MN) (see coding hierarchy in *Extended data:* Annex 4).

### Ethics and reporting

This study received ethical approval from the health research ethics committees of Stellenbosch University (HREC Reference # N18/01/009) and University of Cape Town, and approval from the city of Cape Town to use health facilities in Khayelitsha. We referred to the consolidated criteria for reporting qualitative research (COREQ) to guide the reporting of this study ^17^.

## Results

We conducted 31 interviews between September and December 2018. A summary of the study participants’ characteristics can be found in Table 1.

**Table 1.** Characteristics of study participants (n=31).

*Stakeholder (n)	†Country
Researchers (n=9)	Uganda, Zimbabwe, Malawi, South Africa, United States of America, United Kingdom, Canada, Australia
Clinicians (n=2)	South Africa
Clinician scientists (n=2)	Australia, South Africa
Policy makers (n=1)	Switzerland
Technical agencies (n=1)	Switzerland
Guideline developers (n=3)	India, United Kingdom, Netherlands
Test developers/Industry (n=4)	USA, Germany
Funders (n=1)	USA
Laboratory worker (n=1)	Pakistan
TB survivors/activists (n=4)	South Africa, India
Patients (n=3)	South Africa

* Some participants had multiple roles with regard to TB diagnostics. The stated role was based on the predominant role they discussed during the interview.

† Geographical location was based on their work station or institution at the time of the interview.

**Figure 1.**
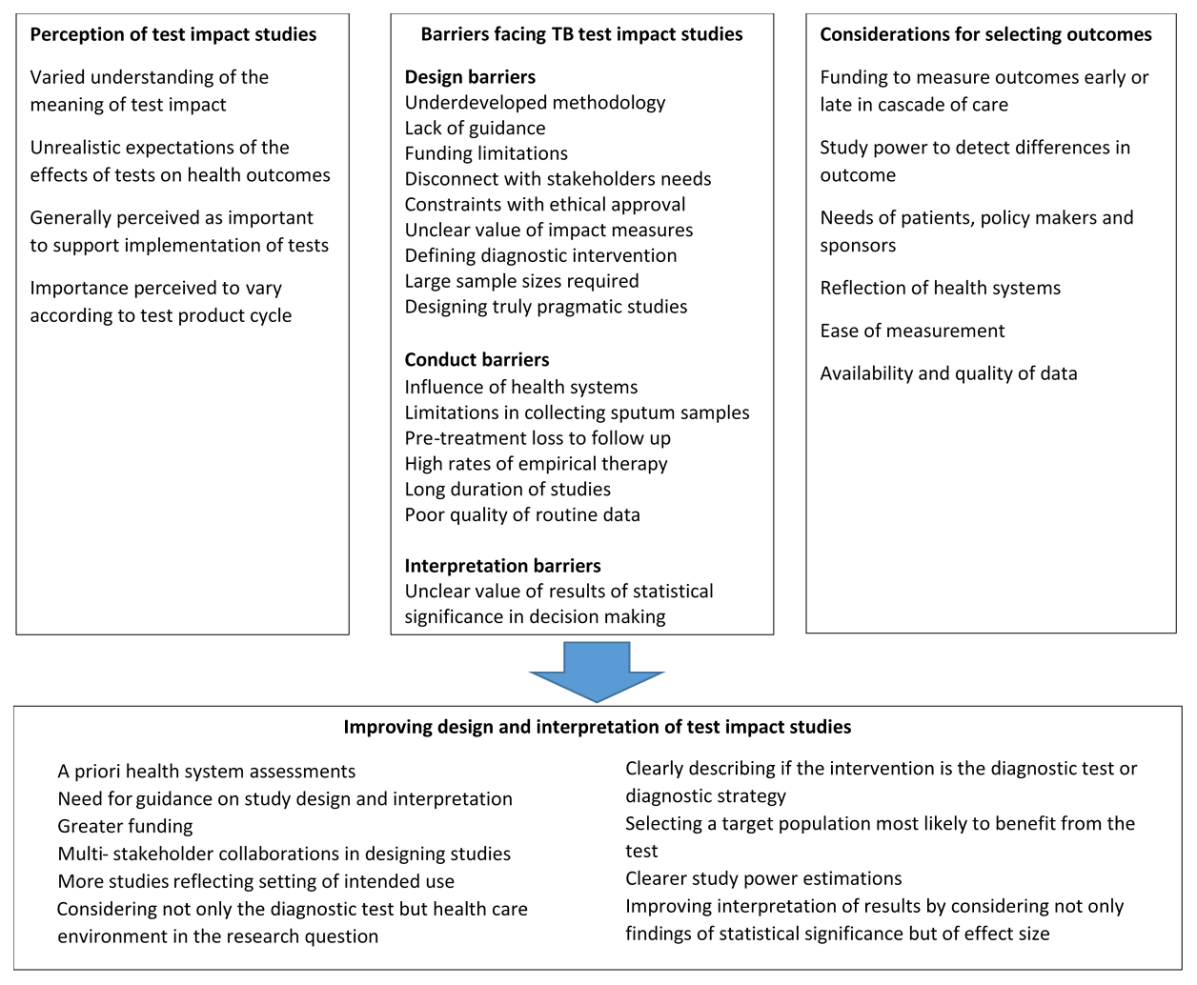
Summary of themes and subthemes about perceptions of test impact studies.

We explored four major themes: General perception of test impact studies, barriers facing test impact studies, selection of outcome measures, and suggestions for improving test impact studies. These themes and related subthemes have been summarized in Figure 1.

### Theme 1: General perception of test impact studies

***Varied understanding of the meaning of test impact***. There were varied responses on the meaning and understanding of what the impact of a test entails. One respondent noted that the term impact is a big term and could mean anything. This response was confirmed by other respondents who stated that their understanding of the term impact of a test included improving health, improving detection of drug-resistant tuberculosis (DR-TB), improving timely treatment through high accuracy and rapid test results, improving access to diagnostic testing and reducing transmission of TB.

*“So, I mean I think impact is a big word right and exactly what we mean by that?”-* (P7, Researcher)

***Unrealistic expectations of the impact of tests on health outcomes***. Some respondents felt that the TB community has unrealistic expectations about the ability of tests to demonstrate improved health outcomes. Contextual factors in the broader health system influence the effectiveness of test rather than a test alone. For example, some felt that the issue limiting the effectiveness of TB tests is not the tests themselves but logistical issues.

*“The technology alone is not the sole determinant of impact, but rather the other supporting elements in the ecosystem that are required to correctly and accurately diagnose patients with TB, and link them to care and on the way to cure”*. – (P20, Funder)
*“So, I think we have the wrong expectations as a community as well.”*- (P27, Technical agency representative)
*“I really look at tuberculosis as a logistics problem.”* – (P13, Test developer)
*“It’s very frustrating when they say with confidence, we need more drugs more diagnostics. No you don’t. You need to make the system work better” -* (P11, Test developer)

***Test impact studies are generally important to support implementation***. Respondents generally perceived that studies evaluating the impact of tests on health outcomes were important. The reasons to support their importance included that they were important mostly to support implementation strategies of tests, and as complementary evidence to test accuracy studies.

* “Generally, I think they are important to support decisions on appropriate implementation strategies for different settings and different countries and with that also decisions on scale up.*”- (P27, Technical agency representative)
*“So the main way we make our guidance and recommendation to countries is really based on the diagnostic accuracy of particular interventions….So these are great studies to complement the other accuracy studies and implementation studies that we evaluate as part of our development of recommendation.”* - (P5, Policy maker)

***Importance of test impact studies depends on product cycle***. Some respondents felt that the need for impact studies depends on the product cycle. For example, an impact study may not be necessary at the beginning when a test has just been developed, due to concerns of delaying market access of the tests. However, it may be necessary after roll out of a test.

*“It is very difficult to use impact information in the beginning to make a decision to invest or not invest and so consequently we are willing to take the risk. Usually in a practical sense to invest or not invest in a technological approach without really understanding the feasibility of ultimately that technology having impact at the other end of the journey”*. - (P20, Funder)

### Theme 2: Barriers facing test impact studies

Barriers facing the design, conduct and interpretation of test impact studies are summarized in Figure 1 and are discussed below.

***Design barriers.***

**Underdeveloped methodology**

Respondents felt that study designs and methods used in test impact studies are still not well developed, hence it is difficult to rely on them to guide decisions on test roll out.

*“So, I think in general it is underdeveloped area….. the overall field of impact assessment is in its nascent state… impact assessments do not feature prominently in that, simply because they are not well articulated in a credible manner so as to provide reliable information to help us make a decision”.* – (P20, Funder)

**Lack of clear guidance**

The lack of guidance for TB test impact studies was discussed by respondents.

*“There was no standard way of doing this, so that was the overall feeling that you just had to come up with whatever you thought was best for the patient so a very subjective view in a way”*. - (P1, Researcher)

**Funding limitations**

Funding was discussed as a major deciding factor of the size and duration of the test impact studies. It was noted that funding to support the studies was often limited.

*“I think maybe it’s many funding institutions do not offer the amount of money you need to recruit thousands of patients and follow then up for years….. If you go to test like every variation of that intervention, you know, you end up having many study arms and rapidly becomes impossible to do the study because it is expensive and will take forever”*. - (P12, Researcher)

**Disconnect in multi-stakeholder needs**

Respondents discussed the difficulties in multi-stakeholder collaboration in the planning and design of multifactorial impact studies.

*“Sometimes you struggle with stakeholder support. I think there’s a bit of a disconnect between levels of government”*. - (P1, Researcher)
*”Well there is a disconnect between what needs to be studied, what is studied, who is being held accountable for the gaps. The diagnostic manufacturers are, often, in my view, being blamed for things that they did not understand were their jobs…” -* (P11, Test developer)

**Constraints related with obtaining ethical approval**

Difficulties in obtaining ethical approval due to ethical concerns of comparing a superior test to standard of care, in resource limited settings, were cited as a barrier to these studies.

*“Is Xpert saving lives?” But at the same time going in front of an ethics committee people, if you want to do a study let’s say of the Omni [novel point-of-care platform for Xpert testing] with the Xpert in comparison with standard of care, which is often still microscopy, people say this is unethical”. -* (P27, Technical agency representative)

**Unclear value of impact measures**

Respondents felt that the value of conducting test impact studies was unclear due to diminished confidence in impact measures used in such studies, and questioned the need to conduct studies for effects that are deemed obvious. They felt that decision makers assumed that an accurate rapid test will obviously improve health outcomes by facilitating early treatment, hence there was no need for impact studies to show that.

*“I think it’s something which the value of doing is unclear… but I also think the confidence that people have and the importance of these impact measures … has begun to be questioned…. So I think, you know, the argument there is it is intuitive and logical that if someone starts treatment earlier there’s probably going to benefit to their health”*. - (P12, Researcher)

**Lack of clarity in defining the intervention**

Respondents cited difficulties in drawing a distinction between a purely diagnostic intervention and other interventions in test impact studies, in light of their multifactorial nature.

*“It’s difficult to draw the line about what their intervention is… if you are testing a diagnostic and then you are also SMS’ing [mobile phone messaging] your result to the patient. And that is not something that happens routinely. Then, you know, obviously your intervention is not just a diagnostic test …. So I think a very hard question to answer is, you know, where will you draw the line or definition of a diagnostic study versus something that is an intervention but includes a diagnostic”*. – (P12, Researcher)

**Large sample sizes**

Very large sample sizes are often required for test impact studies, and these are often not feasible by researchers.

*“I think one challenge is to make sure that there is going to be a sufficient number of people to actually see changes on patient important outcomes”*. – (P19, Researcher)

**Limitations in designing highly pragmatic studies**

In addition, respondents cited the challenges of designing highly pragmatic studies in environments with resource limited health systems.

*“One of the most difficult things to get right is to try and design a study that will assess …what’s going to happen or is happening sort of in real life, pragmatically without influencing sort of routine practice too much, so, pragmatic studies….. your comparison group is always incredibly difficult in real life in sort of pragmatic settings”.-* (P28, Researcher)

***Conduct barriers***

**Influence of health systems**

The influence of, and changes in, health systems on the conduct of such studies, highlighted the multiple factors that face TB diagnostic testing.

*“So at that time we were evaluating Genexpert … which is a really good test but it also depends on other health system constraints, as well (do you have the right staffing) and that’s what ended up happening with Genexpert. At some point we ran out of cartridges and it was difficult to get them to the country. So there are a lot of other complexities but then play an influence in the type of evaluation”.* – (P16, Researcher)
*“There are many changes which occur within the ministries and the health facilities and those may really affect the flow of work that you are doing and those may affect the implementation”.* – (P17, Researcher)
*“So, it’s always very difficult to attribute what is the impact of the actual diagnostic and around that general health system improvements that happened over the same time”.* - (P28, Researcher)

**Challenges in collecting samples**

Other barriers faced during the conduct of these studies were: difficulties in accessing enough samples to evaluate the effect on drug resistant TB, and difficulties in accessing sputum samples in HIV infected people, in children, in very sick patients, and for extrapulmonary TB.

*“I would say that for MTB samples we’ve still have a challenge with resistance samples right and that’s just comes down to the prevalence”.* - (P13, Test developer)
*“Everybody recognises the yield from bacteriological confirmation from young children is low and if people do gastric aspirates, then that is fine and if they can do an induced sputum then that is fine, whatever they are comfortable with, but the yield is low and a negative result, a bacteriological result doesn’t rule out TB. I think it is particularly more important, for example in a project I am working in there is a lot of drug resistant TB including XDRTB, children are contacts and I think we have to try hard in those children to get a sample for Xpert”.* - (P6, Clinician scientist)
*“Because especially when you dealing with HIV infected patients or with paediatrics. The type of sample becomes very difficult”.* - (P16, Researcher)
*“You cannot analyse what impact it has on the extrapulmonary TB case notification”.* - (P8, Laboratory manager)

**Limitations in linkage and adherence to treatment**

Patients and clinicians highlighted challenges with linkage and adherence to treatment in routine health care settings that should be considered when designing TB test impact studies in real life settings. Without efficient processes to ensure good linkage and adherence to treatment, the impact of improved diagnostic tests will be mitigated.

*“So there was no counselling me through it or nothing. I was basically just told that I have multi drug resistant TB and that I need to go to the clinic….So it was scary because I wasn’t really sure what was going on and I was missing school as well so I was anxious about that. I had an idea that it might be TB, but I was very much in denial about it you know… and just the main stumbling block for starting treatment is the disbelief”.* - (P3, TB survivor)
*“The problem is obviously adherence and once they start getting better out of the initial intensive phase, the need to keep coming back and keep continuing their treatment to complete the full course of treatment, adherence can be a challenge”.* - (P6, Clinician scientist)

**Pre-treatment loss to follow-up**

The effect of pre-treatment loss to follow up in diluting the results of test impact studies was discussed.

*“You would find sometimes maybe patients die before they actually get the result of that particular diagnosis”*. - (P16, Researcher)

**High rates of empirical therapy**

The influence of high rates of empirical therapy in diluting the results of Xpert MTB/RIF impact studies were discussed.

*“The findings are diluted by the fact that you have patients who are put on treatment based on clinical suspicion and not necessarily on the diagnostic you are evaluating which almost makes your diagnostic group seem like it’s not working when actually it’s just people are not using it”.* - (P16, Researcher)

**Long duration of test impact studies**

Some respondents felt that conducting test impact studies generally takes longer and would delay the introduction and roll out of new tests to the market.

*“But I think the studies themselves are longer and if everyone waited for that to come out it would only prolong the release of diagnostics”.* - (P13, Test developer)

**Limitations of routine data**

The challenges of routine data in pragmatic studies were also highlighted, including issues with requirements for many approvals to access data, and collecting accurate and complete data.

*“The quality of data is out of your hands usually and it really depends on the system in place and if the health care system has special people in place to collect data and to record it. So what often happens in low resource settings is of course there is no money to hire a special person to collect and record and report data”.* - (P18, Researcher)

***Interpretation barriers***

**Unclear value of results of statistical significance in decision making**

Some researchers questioned the continued endorsement of Xpert MTB/RIF test, despite the lack of statistically significant effects on mortality.

*“We randomized patients to Xpert or not Xpert, and we expected to find a difference and found no difference over and over and over. And yet, despite that, we’ve continued to endorse Xpert…. We’ve now endorsed Ultra, even though all of our studies show that it does not do anything.”*- (P31, Researcher)
*“I think the decision to roll out Genexpert was predominantly a political decision.”*- (P12, Researcher)

### Theme 3: Considerations in outcome selection

Respondents proposed the outcomes they would prefer to be measured in TB test impact studies and commented on the limitations of the proposed outcomes ( Table 2).

**Table 2.** Preferred outcome measures and limitations.

Preferred outcome measures		Limitations
Health outcomes	Clinical impact outcomes Composite outcome around death, hospitalization and undiagnosed TB Contact tracing Cost-related outcomes Culture conversion How to reduce late presentations to clinics Infection control Level of transmission Morbidity Mortality Outcomes measured beyond clinic Patient and health worker safety Prevalence and incidence Quality of life and emotional effects	**Mortality** Mortality is multifactorial and not responsibility of test Mortality limited by downstream effects Mortality limited by long follow up and funding Mortality limited by lost to follow up Mortality limited by poor routine data sources and data availability Mortality limited by study power Mortality may never be zero due to non-TB causes of death
		**Morbidity** Morbidity limited by lack of standard scores Morbidity limited by time and resources
		**Cost** Cost effectiveness preferable at population level not patient level Cost effectiveness does not convey affordability Cost effectiveness don’t consider holistic view Cost effectiveness not relevant unless there is beneficial effectiveness data Cost measurement limited by required resources and expertise Cost proxy measures for patient costs needed where services are free
		**Other health outcome** Onward transmission outcome limited by data Culture conversion does not correlate with transmission Quality of life scores not applicable across cultural contexts
Intermediate outcomes	Acceptability of test and testing process Ease of use of tests Surrogate or process outcomes Test access Test accuracy Time to diagnosis Number of cases diagnosed Number of patients initiated on treatment Time to treatment as preferred outcome Side effects of treatment Treatment success Outcomes in whole diagnostic pathway in different settings	**Intermediate outcome** Test Utilization difficult to measure Time to diagnosis doesn't account for wrong diagnosis Time to treatment not an indicator of completion of treatment

In selecting outcomes as a measure of test impact, the following considerations were put forward by the respondents.

***Funding considerations***. Funding to measure outcomes that can be measured early or late in the cascade of care were considered when selecting outcomes in impact studies.

*“It goes back to that…discrepancy that I mentioned earlier around where…ideally you want to look at the endpoint that is the furthest away. But that’s also the most expensive right? So I, you know, I will put a lot of thought into the study design…I would probably encourage the person to focus on earlier endpoints.”* - (P12, Researcher)

***Statistical power of the study***. The power of the study influences the outcomes that can be measured.

*“We weren’t powered for mortality, so we looked at morbidity, we weren’t powered for culture conversions so we looked at time to treatment”*. - (P12, Researcher)

***Patient needs***. The selection of outcomes that reflected needs of patients such as timeliness of care (time to diagnosis and time to treatment) and impact of patients’ health such as morbidity and mortality were mentioned.

*“Help me today. Diagnose me today. Start me with treatment today.”* - (P14, TB survivor)
*“I do think the patient oriented outcomes are important and I think we should be looking at mortality and morbidity and not just the simple diagnostic accuracy, sensitivity, specificity”.* – (P7, Researcher and guideline developer)

***Sponsor and policy maker needs***. Some respondents mentioned how study sponsors decided the outcomes of the studies *a priori*, and others discussed how the political agenda determined the outcomes decision makers would be interested in.

*“The first thing that guided it was the study’s sponsor really. I am talking about one in particular where the study’s sponsor determined that they wanted mortality, despite a big study …. showing that Genexpert did not reduce mortality”.* - (P1, Researcher)
*“I think the impact metric that policy makers want to use is what suits the political agenda”*. – (P12, Researcher)

***Reflection of functioning of the health system***. Intermediate (also known as surrogate) outcomes such as time to diagnosis were discussed as suitable for demonstrating the functioning and quality of the health system, and would thus inform roll out of tests.

*“I’m a strong believer honestly, in surrogate outcomes simply because I think it holds the diagnostic tests to the bar of the whole health system”.* – (P27, Technical agency representative)

***Ease of measurement***. The ability of an outcome measure to give unequivocal or unambiguous results such as mortality, and ease of measurement such as time to diagnosis, and time to treatment were considered in selecting outcomes. For example, mortality can easily be assessed because patients can be traced, and death can be recorded. Quality of life measurements were preferred by some, because standardized scores or widely accepted tools for measuring them exist. Morbidity was regarded as difficult to measure because of lack of standardized scores (see Table 2).

***Availability and quality of data***. The availability and quality of data was an important consideration when selecting the outcome to be measured. Respondents stated that analyses of outcomes such as ongoing transmission of infection were limited by availability of data. In routine settings especially, assessment of mortality would be limited by loss to follow-up and poor routine data sources (see Table 2).

### Theme 4: Suggestions for improving impact studies

Respondents suggested the following areas for consideration in improving impact studies;

***Health system assessments prior to study***. Many respondents stressed the need for a thorough systems evaluation, or process evaluation, prior to conducting test impact studies. This would guide the design, conduct and interpretation of the impact studies, and aid more accurate attributions of a test’s impact.

“ *Well, before they do it they should comprehensively do a systems analysis and there are a lot of ways to do this. I think the patient cascade of care and the patient pathway should be accurately dissected so that in the journey of the patient from seeking healthcare to cure, that all the things that could derail, assuming you have a perfect diagnostic test, all the other things that could derail that cure should be examined because at the end of the day a diagnostic test alone has zero impact and it simply creates a result and the question is how does the system deal with that result and how does it affect the actual delivery of care.”*- (P20, Funder)

***Greater guidance and support***

**Availing guidance for study design**

The need for standardized guidelines on how to design, conduct and interpret such studies was suggested.

*“If you have a clear guidance and a specific description and a standardisation that would be, I think, very beneficial, a beneficial guide in order to follow, so that everybody knows exactly what to do.”* – (P23, Test developer)

**Need for greater funding**

Some respondents advocated for more resources to conduct complex impact evaluations of TB diagnostics.

*“…. and the fact that they are quite difficult evaluations to do… So the fact that we are taking interest in this field is quite obviously that it is a major problem… So I think if you are prepared to take it on then everyone is very interested in the results…..And is the funds are easy to get hold of if you make a good case.”* - (P15, Researcher)

**Strengthening multi-stakeholder collaborations and support**

Respondents suggested greater collaboration between producers and users of research to provide evidence that was truly useful to end users. They also stressed the need for collaboration at all levels of health systems governance from the beginning of the study, in order to account for all factors that could influence test impact studies.

*“It is quite an enabling factor in that you bring on board all types, you bring on board the time when we are doing our proposals and share our ideas and discuss with them and then at the end of the day, we are sure that they have participated their ideas have been incorporated…”*- (P17, Researcher)

***Improving study design***. Respondents suggested various aspects to be considered when designing test impact studies, including: improving study design by proposing diagnostic questions that consider the broader health care ecosystem, and measuring interventions that include the diagnostic test and accompanying clinical information, selecting the target population at the health care level most likely to benefit from the test, and setting realistic targets for outcome reduction in the sample size calculations.

*“These are not the right questions. The question is can it perform in this environment? And so I think doing an impact study on usability at different levels of the health care system is central information for buying and fitting your country with the right diagnostics.”-* (P11, Test developer)
*“Make sure you’re measuring the right intervention, which is using all the information available to you, not just blindly doing a test without taking into account the relevant clinical information which is there which is fully available.”*- (P31, Researcher)
*“I think the only place to clearly demonstrate the impact on patient’s important outcomes tends to be when diagnostic tests are used among the sickest patients…. If you want important outcomes, then you have to be very selective about the population that you are going to study”.* - (P19, Researcher)
*“And, you know, to be realistic about what you were expecting. You often see studies where there’s quite unrealistic targets set in the sample size. So, it has to be well powered. It has to be large and you have to pick a patient group that you think has got the most to benefit from it.”* - (P15, Researcher)

**Need for more highly pragmatic studies**

To enable decision making some respondents stressed the need for such studies to be designed and conducted in settings of intended use.

*“The impact assessment needs to be done in the setting of intended use on the target population for which the intervention is likely to have the greatest impact.”* - (P5, Policy maker)

***Improving interpretation of study results.***

**Considering the magnitude of absolute reduction in interpretation**

Improving the clarity on the implications of statistical significance on decision making by focusing not only on statistical significance but the magnitude of reduction was discussed.

*“The reduction in mortality of Xpert across an individual patient meta-analysis is twelve percent but it’s just not meeting statistical significance, but it’s twelve percent absolute reduction.”*- (P27, Technical agency representative)
“ *….There’s evidence of absence, but that’s not the case, there’s simply absence of significant evidence, but there’s enough evidence to actually show that it is substantially impactful on surrogate outcomes and substantially impactful on the very important outcomes, such as mortality and it’s just not statistically significant… but then it comes back to the question that if you don’t show effect, it’s not necessarily the fault of the test and it doesn’t necessarily mean we should not roll out this test*.”- (P27, Technical agency representative)

## Discussion

Our study explored the perceptions of different stakeholders about studies evaluating the effect of TB diagnostic tests on health outcomes, and identified suggestions for improving these studies. In summary, our study showed that stakeholders had different expectations with regard to test impact and how it is measured. TB test impact studies were perceived to be important for supporting implementation of tests but there were concerns about the unrealistic expectations placed on tests to improve outcomes in health systems with many influencing factors. To improve TB test impact studies, respondents suggested conducting health system assessments prior to the study; developing clear guidance on the study methodology and interpretation; improving study design by describing questions and interventions that consider the influences of the health-care ecosystem on the diagnostic test; selecting the target population at the health-care level most likely to benefit from the test; setting realistic targets for effect sizes in the sample size calculations; and interpreting study results carefully and avoiding categorisation and interpretation of results based on statistical significance alone. Engaging multiple stakeholders when designing these studies, advocating for more funding to support robust studies and conducting more highly pragmatic studies were also suggested.

Expertise and role in the health care system contribute to how test impact is perceived and measured ^5^. To improve the usefulness of results to end-users, researchers designing the impact studies need to seek insights from various stakeholders involved in decision making about TB diagnostic tests. This will clarify which patient-important outcomes are considered important at the study design stage.

Qualitative research exploring the complex process involved in impact evaluations of TB tests is scarce ^13^. Existing qualitative studies about TB diagnostic tests focus mainly on stigma and disease perceptions influencing diagnosis ^13, 18– 22^, barriers facing TB evaluation services, or TB control efforts and factors influencing delays in TB diagnosis ^15, 23– 26^. These studies nonetheless give insight on the health system barriers that may affect the implementation of TB diagnostic tests, and indirectly flag aspects that researchers ought to consider when designing and conducting TB implementation trials in routine settings. For example Cattamanchi and colleagues ^25^ demonstrated that health system barriers (stock outs, limited infrastructure, poor staff motivation, high workload, poor coordination of health services) and setting barriers (stigma, patient time and costs) both impede TB diagnosis, and if not addressed could impede TB case detection. Indeed, one respondent in our study cited stock outs in Xpert MTB/RIF cartridges as a challenge that delayed their impact study. Unavailability of tests could contribute to high rates of empirical therapy in a study, mitigating the effect of Xpert on mortality.

Since the initial recommendations for the use of Xpert MTB/RIF in 2010 ^27^, we still lack strong evidence of the test’s impact on people important outcomes ^28^. Calls have been made to better understand how to implement and evaluate this test (as well as the newest version, Xpert Ultra) in weak health systems ^28, 29^. The effective implementation of Xpert MTB/RIF has been limited by funding, lack of comprehensive diagnostic implementation plans, evaluations suggesting limited impact and weak health systems ^29– 31^. The design and execution of implementation trials evaluating the effect of Xpert MTB/RIF (and Xpert Ultra) on health outcomes thus needs to consider the health ecosystem in which the test is expected to perform ^28, 30, 32^. This could be done by incorporating process evaluations ^33, 34^ before or alongside the trials to understand the different diagnostic implementation processes, and how the diagnostic interventions and the health ecosystem interact with each other in the TB cascade of care. Qualitative research methods ^33^ incorporated in these process evaluations can explain how interventions work, why interventions do not work, and explore factors influencing the delivery and implementation of an intervention. Process evaluations have been used to inform the design of trials evaluating the impact of malaria diagnostic tests ^35, 36^. For example, Ansah and colleagues ^35^ evaluated the impact of malaria rapid diagnostic tests on fever management in Ghana. To inform their study design they conducted a baseline study of available antimalarial drugs and also conducted focus group discussions to explore the acceptability of their intervention and how best to introduce it.

The updated recommendations on the use of Xpert MTB/RIF advised that impact evaluations be done, but did not provide detailed guidance on how to do so ^30, 37^. To design effective implementation trials and impact evaluations, guidance informed by programmatic data specific to real life settings is needed. The impact assessment framework for TB diagnostic tests proposed by Mann and colleagues ^38^ discussed areas and different types of analyses (effectiveness, equity, health systems, scale-up and policy analyses) to be considered in impact assessments in general. This framework was however not specific to trials or studies evaluating the impact of TB diagnostic tests on health outcomes. Schumacher and colleagues described the range of study designs that can be used to assess the impact of TB diagnostics but did not provide guidance on how to conduct such studies ^6^. Guidance on designing effective impact trials of TB diagnostic tests could address areas highlighted in the findings of our study including how to use a priori process evaluations to guide the design of impact studies, and how to improve the study design by defining the diagnostic intervention, setting realistic targets in sample size calculations, selecting appropriate target populations, and guiding the selection of outcomes to be measured. Such guidance could also suggest how to incorporate the views of different stakeholders in the design and conduct of the impact studies and offer direction on how these studies can best be interpreted.

Our study had a number of strengths. We incorporated views from various stakeholders, including patients, to obtain a holistic view of the multi factorial components of test impact studies and we followed the COREQ guidelines in reporting our study. Our study was however limited by the fact that we interviewed only those who responded to our invitations. Participant bias where respondents give expected and socially desirable answers could also have occurred. We tried to mitigate this by asking open ended questions. Most stakeholders interviewed were based in high-income countries, or from India and several countries in Africa including South Africa, Malawi, Zimbabwe and Uganda. This could limit the applicability of our findings. Most respondents gave their perceptions about Xpert MTB/RIF. We did not explore perceptions of studies evaluating the effect of a point of care urine based lipoarabinomann assay (LAM) on health outcomes. Trials evaluating the impact of this test have also shown variation in effects on health outcomes with some demonstrating conclusive reduction on mortality ^39^ and others inconclusive effects when TB LAM ^40^ is compared to standard of care. Nonetheless, the effect size for LAM in those trials was about a 10-20% mortality reduction similar to Xpert MTB/RIF ^7, 8, 39, 40^. Perceptions and insights explaining the significant effects of the TB LAM test would also be useful in guiding the design of impact evaluations of novel TB diagnostic tests.

In summary, TB test impact studies were perceived to be important to support implementation of tests but there were concerns about their complexity and how they are influenced by the health system context. Process evaluations of their health system context and guidance for their design and interpretation are recommended.

## Data availability

### Underlying data

Ethical approval from the ethics committees and informed consent by participants was granted to disseminate de-identified data. Relevant de-identified quotes to support the results provided have been included in the main manuscript. Despite de-identification, transcripts of the interviews have not been provided because information contained in the transcripts can betray the identity of participants. Any further requests for particular de-identified data or quotes can be granted by contacting the corresponding author directly.

### Extended data

Open Science Framework: Qualitative study: Improving the design of impact studies for TB diagnostic tests”, https://doi.org/10.17605/OSF.IO/GRY2N ^41^.

This project contains the following extended data:

- Annex 1_Invitation letter.docx
- Annex 2_Topic guide.docx
- Annex 3_Patient Consent forms-(english_isiXhosa).docx
- Annex 4_Coding and theme hierarchy_Final.docx

### Reporting guidelines

Open Science Framework: COREQ checklist for Improving the design of studies evaluating the impact of diagnostic tests for tuberculosis on health outcomes: a qualitative study of perspectives of diverse stakeholders, http://doi.org/10.17605/OSF.IO/GRY2N ^41^.

Data are available under the terms of the Creative Commons Zero "No rights reserved" data waiver (CC0 1.0 Public domain dedication).
