# Supplementary material for: Improving the design of studies evaluating the impact of diagnostic tests for tuberculosis on health outcomes: a qualitative study of perspectives of diverse stakeholders
Source: Wellcome Open Res. 2019 Nov 21;4:183. [Version 1] doi: 10.12688/wellcomeopenres.15551.1 (PMC7041361; doi:10.12688/wellcomeopenres.15551.1)
Supplement: Peer review history

# Peer review: Reviewer response for version 1

**DOI:** 10.21956/wellcomeopenres.17029.r37642

**Published:** 2020-02-19

**Authors:** Elizabeth E. Lutge

**Affiliations:** 1 University of KwaZulu-Natal, Durban, South Africa

This was an interesting article, the research was well conducted and the conclusions rooted in the data and analysis. However, there were one or two areas which I felt could have been improved upon.

 The sampling of participants was, as the authors acknowledge, limited because only those who responded to the invitation were included in the study. Another potential bias which they do not discuss, is the preponderance of researchers in their sample. One of their aims was to obtain maximum variation in categories of respondents, and although this was achieved to a certain extent, the higher number of researchers in their sample may have biased their results to the researchers’ points of view. In addition, although they included patients in their sample, the views of patients were reported on much less than those of other respondent categories, leading to a narrowing of the focus of this paper. Whilst this in itself is not a bad thing, it does undermine to some extent the achievement of the holistic view that the authors were aiming for.

 A second area, which could be improved even though the study is complete, is the consideration of the more general issues of how research questions are generated, how research projects are formulated, and how stakeholders interact to ensure that research is useful to as wide a range of stakeholders as possible. A key concern of this study was how research on TB diagnostic tests fails to meet the expectations of stakeholders. This is an important concern around research in general, and the authors could draw on this literature both in the contextualization of this study, as well as in the discussion of their results.

I confirm that I have read this submission and believe that I have an appropriate level of expertise to confirm that it is of an acceptable scientific standard.

# Peer review: Reviewer response for version 1

**DOI:** 10.21956/wellcomeopenres.17029.r37140

**Published:** 2020-01-31

**Authors:** Andrew McDowell

**Affiliations:** 1 Department of Anthropology, Tulane University, New Orleans, LA, USA

This article provides an important window on the production of public health, scientific, and technological consensus. It provides readers a view on the concerns of various actors within the TB cascade of care and public health bureaucracies the world over. It helps readers think about the priorities used when designing and analyzing test evaluations and begins an important discussion about the difference between positive evaluations and positive health outcomes for patients. It has a sufficiently large sample and presents a variety of perspectives on evaluation and implementation of two molecular tests for TB. As such it merits indexing. However, the paper has a few areas in which improvement is possible. I suggest the authors consider them as they revise the paper and design future work. They are as follows:

**Introduction**: 

 The paper asserts that there is ‘uncertainty about the effects of Xpert… on people-important outcomes.’ It is not clear what defines a people-important outcome. Do non-people-important outcomes exist? If so, what are they? Why are these non-people important outcomes important measures of success if people are not being reached by these technologies and lives not saved. The paper as a whole is challenged by a seeming disjuncture between the saving of lives and the perpetuation of global health expertise. To my mind this is a symptom of broader issues in global health, but I would caution the authors to avoid perpetuating such a descriptor. People important outcomes are the central point of our collective endeavor, not a subset. 

 The final two paragraphs of the introduction suggest that the paper will help readers better understand the effects of “perceptions of participants who are part of TB diagnostic studies, and help shed light on why and how these tests work in different contexts, and on how best to implement them.” To my understanding the paper suggests, then, that we must learn how to implement diagnostic tests from their designers. This claim is widely refuted in qualitative literature produced by the field of science studies which suggests that implementation must be understood from the user and the designer’s perspectives (see Madeline Ackrich among others). If this is the argument of the paper, it must refute an entire well established discipline. That seems a tall order. 

 The paper quickly shifts to argue that studying test designers and diverse stake holders will help address problems with those studies. Though this seems useful, it’s not clear what makes this a publishable finding. Surely, scientists talk to each other and discuss how they evaluate studies all the time. Is this currently not happening for TB? If not, why not? If so then why is this paper doing anything more than asking colleagues for their suggestions? It’s unclear how interviewing the creators of studies evaluating impact will affect the impact of a diagnostic test. The authors seem to be watching the bee-watcher and suggesting doing so will tell us about the bee. If that’s the case, they must make a stronger argument about why. How can improving evaluation lead actual improvement in performance at the health system level and for our patients? 

**Methods**:

 First, the study is not phenomenological. It is descriptive. It does not fit into the broader history and philosophical school of phenomenology and this term is inappropriately used here. Descriptive is best. One could also argue that the study falls somewhere in an interactionist paradigm but perhaps that’s not of much use here. Finally, the paper, like all papers cannot attain a complete description of anything. This modifier should be stricken during revision. The paper tells us many things and should be lauded, but it is important to be both realistic and modest at the same time. 

 The authors also tell readers that they considered maximum variation with regard to expertise, but do not tell us how they did so or how they define expertise. How were people put in categories? Don’t many of these categories overlap. It seems that the sample is very heavily weighted to researchers and one wonders why. What effect might this have had on results? It could even be a positive effect. 

 It strikes me that the interviews were rather short. Why was this the case? How might their limited time span effect data quality? Might this be a limitation? 

 The authors should add a paragraph describing the non-coding analytic work they did. Coding is a process of preparing data for analysis, not for analyzing it. What kind of analytical work has been done here? This revision is essential. Often in qualitative research analysis is looking for connections, considering outliers, or looking for variation of a transversal theme based on interviewee positionality. Did the authors do any of this work? 

**Results**:

 The results say that authors explored four major themes. Were these themes designed into the interview process, or did they emerge from the interview? Both ways accessing information are valuable, but it is important to clearly state the way you came to these themes. 

 The final sentence on column 1 on page 5 refers to a TB community. It’s not clear what ‘the TB community’ is. Some precision here would be useful. Certainly, patients - the majority of the TB community at a planetary scale - would not be particularly concerned about a test’s ability to demonstrate improved outcomes. I’d suggest redefining the term here to represent the small network of actors who make decisions about tests and their expectations. In this same paragraph, the authors suggest that logistical issues thwart test effectiveness. This has little to do with the paper’s stated purpose of better assessing test impact. How will this data point help the better assess a tests’ potential and actual effects? It seems essential but the paper is unable to effectively develop in it as written. 

 Table 2 in the results section could be more clearly referenced to in the text. It is not immediately clear what the differences between “health outcomes” and “intermediate outcomes” are. It’s also not clear who is doing the preferring in “preferred outcome measures.”

**Discussion**:

 The discussion’s first line is symptomatic of a larger question that the publication of this study poses. It reads, “Our study explored the perceptions of different stakeholders about studies evaluating the effect of TB diagnostic tests on health outcomes…”. The clear statement of purpose here gestures to a conflation made by the authors. They write assuming that better evaluation of tests would result in better outcomes. On what data do they base this first principle. They follow this sentence with a second contradictory sentence a few lines later: “TB test impact studies were perceived to be important for supporting implementation of tests but there were concerns about the unrealistic expectations placed on tests to improve outcomes in health systems with many influencing factors.” Indeed this claim undercuts their methods and purpose of the study as well as the first quote in the section. Nonetheless the argument it is strongly supported by the data they present in the results section. This, to my mind, indicates the importance of studies that move past questions of how to better assess those that ask how do we know, predict, and anticipate health systems when designing and anticipating the effects of a test. The paper is unable to access this information due to its sample. Still, these questions are crucial and might be gestured to in the conclusion. 

 The authors suggest that qualitative research on impact evaluation is scarce. Though this claim is true, there is a large body of anthropological and STS literature on randomized control trials, test development, and global health metrics. I encourage them to look to work conducted in Oslo, Maastricht, Edinburgh, and Berkeley among others. It is also not totally clear to this qualitative scientist how epidemiologists with a three month course in qualitative methods feel comfortable making such broad claims about qualitative research. Their paper is a good one and it has a valuable store of important information, but perhaps calls to qualitative research action and broad claims about the need including qualitative methods in process evaluation might best be left to others. Even this paper does not incorporate qualitative methods in processes evaluations, though I would encourage authors to do so. 

 This points to a need to better sum up what the paper does tell us rather than make larger claims about where and what research ought to do. The paper indeed shows that “TB test impact studies were perceived to be important to support the implementation of test but there were concerns about the complexity of how they were influence by the health system context.” This is a central finding and I’d encouraging reconfiguring the discussion around this claim. It may help authors resolve the problems created by their tacit assumption that better evaluation will solve structural problems. To my mind this is not the case; but designing evaluation with structural limitations in mind and developing new ways to account for them when evaluating the possible effect of a diagnostic is essential. This is, after all, the task the authors set themselves out to do. I encourage them to re-configure the discussion to do attend to such considerations and help evaluation designers think innovatively about how context does and will always matter. 

 Though this piece has limitations, it is a crucial step in recognizing that accounting for context is essential when conducting evaluations and predicting effect. It begins an important conversation about the centrality of considering the context in which tests are being used and how they may affect the care and system related effects of a technology. It could be an important tool for improving the quality of TB care and reminding us that a test not connected to care, despite many favorable evaluations, has squarely achieved failure.

I confirm that I have read this submission and believe that I have an appropriate level of expertise to confirm that it is of an acceptable scientific standard.
